# COVID-19 and alternative assessments in higher education: implications for academic integrity among nursing and social science students

**DOI:** 10.1007/s40979-023-00129-0

**Published:** 2023-05-01

**Authors:** Esther Abena Adama, Amanda Graf, Kwadwo Adusei-Asante, Ebenezer Afrifa-Yamoah

**Affiliations:** 1 School of Nursing and Midwifery, Edith Cowan University, 270 Joondalup Drive, Joondalup, WA 6027 Australia; 2 School of Nursing and Midwifery, University of Notre Dame Australia, 19 Mouat Street, Fremantle, Western Australia; 3 School of Arts and Humanities, Edith Cowan University, 270 Joondalup Drive, Joondalup, WA 6027 Australia; 4 School of Science, Edith Cowan University, 270 Joondalup Drive, Joondalup, WA 6027 Australia

**Keywords:** Assessments, Academic misconduct, Cheating behaviours, Tertiary students, University education

## Abstract

**Background:**

COVID-19 and its associated restrictions called for innovations in higher education teaching and learning space with many universities resorting to online teaching and alternative assessments. However, little has been done to understand the academic integrity implications in alternative online and non-invigilated assessments.

**Aim:**

This study explored the perceptions of higher education students regarding academic integrity in alternative assessments.

**Methods:**

Cross-sectional mixed method design following the parallel convergent approach was utilised in this study. A convenience sample of 380 undergraduate and postgraduate nursing and social science students completed an online survey on academic integrity behaviours associated with alternative assessments.

**Results:**

High risk (31.7%) of academic misconduct was perceived among young people (18-24 years old). Collusion was common among nursing students (24.5%) and cheating likely to occur in assessments with longer duration—between 2 and 4 hours (18.8%) and between 1 and 2 weeks (46%). Qualitative data resulted in 274 findings and three themes— (i) impossible to cheat; (ii) easy to cheat and (iii) understanding the consequence of cheating. Suggestions for preserving academic integrity in alternative assessments were also made from the qualitative data.

**Conclusion:**

Like other forms of traditional assessments, alternative assessments have increased risk of breach of academic integrity; however, with the right strategies, they could serve as effective means of assessing learning outcomes.

**Supplementary Information:**

The online version contains supplementary material available at 10.1007/s40979-023-00129-0.

## Introduction

Over the years, higher education has been responsive to global issues. In 2020, when COVID-19 was declared as a global pandemic by the World Health Organisation (World Health Organization 2020), all aspects of life, including higher education, were affected (Aristovnik et al. 2020; Toquero 2020). To reduce the spread of the COVID-19 virus, many restrictions including social distancing were put in place. As a result of these restrictions, higher institutions of learning in 185 countries were closed (Marinoni et al. 2020). To prevent the long term impact of closing institutions of higher education, many technological innovations such as online teaching and assessments became the norm for both developed and developing countries (who are less technologically rich) around the world (Adnan and Anwar 2020; Paudel 2020). Although online and non-invigilated assessments are associated with challenges (Alruwais et al. 2018; Guàrdia et al. 2017), they became the global norm during COVID-19 pandemic and may be considered in higher education to curb the potential for academic misconduct associated with sophisticated Artificial Intelligence applications.

Prior to COVID-19 pandemic, higher education has been striving to ensure assessments are fit for achieving learning and graduate outcomes (such as employability or industry readiness) with recommendations to move from test or examination-based assessments to assessments that test higher order cognitive skills (Bloxham and Boyd 2007; Merta Dhewa et al. 2017). Bloxham and Boyd (2007) argue that although examinations are widely used to assess learning outcomes, their disadvantages far outweigh their advantages. Advantages such as reduction of risk of cheating, testing knowledge and increasing understanding of concepts during end of semester revision were reported. On the other hand, disadvantages of examinations/test such as inferior/superficial quality of learning, anxiety provoking, lack of continuous learning (leaving learning to end of semester), promoting memorisation and limiting originality and restriction of opportunity for real-world learning have been explored (Bloxham and Boyd 2007). Although examinations/tests are highly criticised for assessing knowledge and understanding at a superficial level, they can still be used in certain assessments where memorisation is required.

In response to the COVID-19 global pandemic, Australian institutions of higher education, like many countries, rose to the challenge of online teaching and assessments (Bozkurt et al. 2020; Gamage et al. 2020; Reedy et al. 2021). Traditionally, in the pre- COVID-19 era, many end of semester assessments were invigilated/proctored examinations whereby students are required to gather in examination halls and complete assessments within a specified period. Although the strict invigilated examinations caused high levels of anxiety and stress for students (Clutter et al. 2017), it is perceived to preserve academic integrity as opposed to online assessments (Johnson 2018). However, due to COVID-19 restrictions, invigilated examinations were replaced by alternative assessments via various online platforms, where students completed their semester assessments remotely without a proctor.

Alternative assessments became widespread and undertaken throughout the year 2020 as a measure to reduce the spread and manage uncertainties of COVID-19 virus. Alternative assessments comprise various types of assessments such as oral assessment or VIVA via Zoom or similar video conferencing, submission of video recordings of presentations, live psychomotor skill demonstration via Zoom, take-home open book assessments, time-limited online open-book assessments and assignments. Take-home long and short answer assessments were completed in Cadmus (an online assessment platform) or submitted via Turnitin. Using real-world scenarios, the alternative assessments were designed to assess learning outcomes and higher order capabilities such as critical thinking, reasoning, problem solving, skill acquisition, synthesis, conceptualisation, evaluation and communication skills in order to reduce or eliminate the chances of academic misconduct (Guàrdia et al. 2017; Roksa et al. 2016).

For years, online assessments are perceived as being associated with high risk of cheating or academic dishonesty (Costley 2019; Harmon et al. 2010; Kocdar et al. 2018). However, interventions such as appropriate design of assessments that are aligned to learning outcomes; oral assessments to complement online examinations and maintaining culture of academic integrity in higher education have been cited as means of preventing academic dishonesty in online assessments (Akimov and Malin 2020; Harmon et al. 2010; Peterson 2019). Based on experiences of academics and current evidence on assuring academic integrity in online or non-invigilated assessments, the alternative assessments were designed based on current best practice to prevent or reduce the risk of academic misconduct. Relative to the research setting, some strategies were implemented to safeguard academic integrity for the alternative assessments. They include development of problem-based or application of theory to practice questions, presentation of photo ID for all the VIVA’s, a five-minute video providing ID and explaining one answer from the take home assessment, the assessor marked the video alongside the written assessment. Cadmus and Turnitin plagiarism detecting tools were also utilised for the various alternative assessments.

Given the importance of assuring academic integrity and the role of assessments in achieving learning/graduate outcomes in higher education, it is imperative to explore the students’ perceptions of alternative assessment methods in order to gain insights into possible academic integrity issues associated with alternative assessments in higher education. Therefore, at the end of the 2020 academic year, we explored students’ perceptions of academic integrity issues associated with alternative assessments.

## Methods and materials

Cross-sectional mixed method design was used for this study. Cross sectional research is effective for gaining a snapshot from a given timeframe (Wang & Cheng, 2020). Surveys are applicable for data collection used in a cross-sectional study. The mixed method approach in this study followed a parallel convergent pattern. Both statistical and narrative views are considered when applying a mixed method approach (Creswell, 2017; Tashakkori, Johnson, & Teddlie, 2020). Strengths of convergent mixed method design consists of it being efficient, collecting both qualitative and quantitative data at the same time, and allowing for the same viewpoint to be obtained (Creswell & Clark, 2017). Creswell and Clark (2017) highlight that the method allows for the analyses to occur separately, and comparisons of inferences occur before merging the information throughout the discussion.

A self-reported online questionnaire (Qualtrics powered) was developed based on various evidence on higher education teaching, assessment and academic integrity behaviours (Adesile et al. 2016; McCabe and Trevino 1993; Ramdani 2018). The survey consisted of both quantitative and qualitative questions; it was made up of 22 items expected to be completed within 15-20 minutes. It consisted of both 5-point Likert scale (1-strongly disagree to 5-strongly agree) on self-reported and perceived academic integrity statements and open-ended questions to provide further information on Likert scale responses. This paper presents an aspect of a larger study that investigated students’ perception of alternative assessment on their learning, academic integrity and mental health during the pandemic. The cross-sectional study explored the perceptions of students regarding the alternative assessments delivered during the 2020 academic year as a result of COVID-19 restrictions (Additional file 1).

To ensure the rigour of the data collection tool, the face validity of the research questionnaire was reviewed by two experts in higher education in the two disciplines. The Associate Dean of Teaching and Learning in both disciplines reviewed and provided feedback on the survey instrument. The feedback was then incorporated into the survey and piloted with four students within one undergraduate course to ensure comprehension and appropriateness of the questions (Bowden et al. 2002) while assessing both face and content validity. Thereafter, appropriate revisions were made to the survey questionnaire before data collection.

### Participants

The participants invited to complete the survey were from two schools (School of Nursing and School of Arts and Humanities), across stages within their courses in a public university in Australia. Students who have undertaken alternative assessment in the 2020 academic year were invited to complete the online survey. The survey was available from August to December 2020 for both domestic and international undergraduate and postgraduate students. Students who completed the pilot survey were excluded from the main study. In all, 380 participants completed the survey.

### Ethical consideration and data collection

Ethics approval was obtained from the prospective university’s Human Research and Ethics Committee (2020-01533-ADAMA). The study information sheet and informed consent were embedded in the survey link and a forced response applied. All students provided consent prior to completion of the survey.

Convenience sampling was applied as all undergraduate and postgraduate students across two schools and multiple courses were invited to complete the survey. An invitation email was sent to students by the course/programme directors and associate deans of teaching and learning of the two schools to avoid potential for power-imbalance and conflict of interest between students and teachers (authors) and to encourage voluntary participation in the study.

### Measures

#### Demographic data

Biographic data included age and level of education, programme/course of study and number of subjects/units enrolled in one semester. To ensure confidentiality, students’ identity data were not collected.

#### Academic integrity measures

We used academic dishonesty behaviours (or behaviours that were discouraged during the assessment) such as seeking help from peers, collaborating with others and seeking help from experts/ professionals/ senior colleagues to describe academic integrity concerns. These behaviours were presented on a Likert scale and students agreed or disagreed with them.

#### Qualitative measures

Open-ended questions were included to expand on the responses from the Likert scale. These also provided participants the opportunity to share their experiences with academic integrity issues associated with the alternative assessments. Participants responded to the specific open-ended question “Do you think the alternative assessment made it easier for students to cheat? Please explain”.

### Data analysis

#### Quantitative data analysis

Statistical analysis of quantitative data was carried out in SPSS version 26. The quantitative segment evaluated the relationship between demographics (age, course, level and stage of study, and number of units enrolled) and academic integrity measures, namely, seeking help from peers, collaborating with others, and seeking help from experts/ professionals/ senior colleagues. Frequencies and percentages highlighting the distributions across levels of the demographics for the measures were then reported. The chi-square test was carried out to establish significance or otherwise of these relationships. A logistic regression was formulated to further quantify any differences that may exist at the various categorical stratification or segmentation of our study participants.

#### Qualitative data analysis

**Fig. 1:**
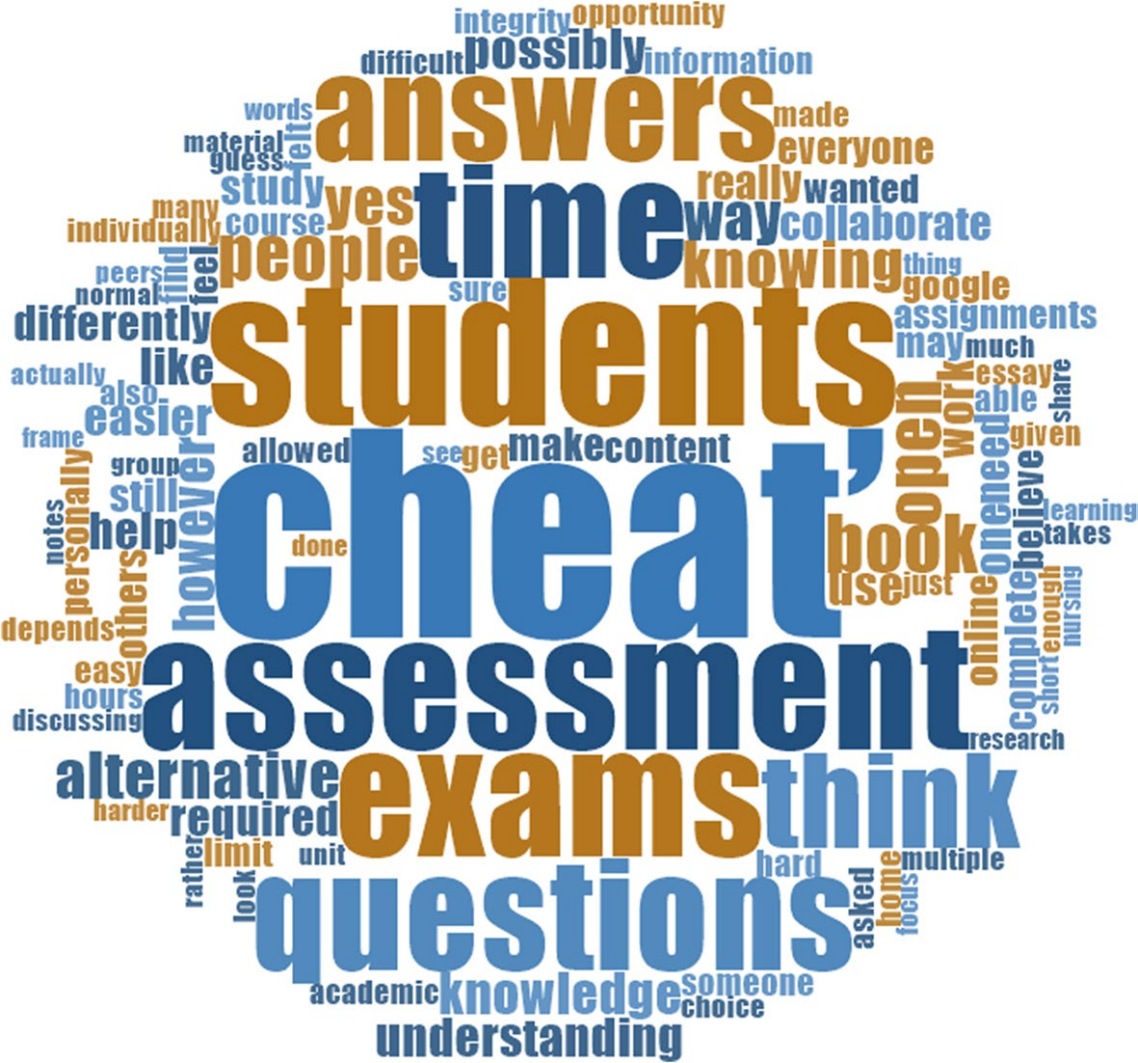
Overview of qualitative responses via word cloud

Thematic deductive and inductive data analysis of open-ended questions was undertaken in NVivo 12 to generate codes that represent students’ perceptions of academic integrity in the alternative assessments (Feng and Behar-Horenstein 2019). Deductive analysis was guided by the research questions and inductive analysis undertaken by reading the raw data back and forth and immersing in the qualitative dataset. Word Cloud visualisation was also used to show the word frequency within the qualitative data (Fig. 1).

## Results

### Quantitative findings

The modal age bracket of the respondent was 18-24, and mostly pursuing undergraduate degrees (83.9%). Majority of the respondents were first (29.2%) and second (34.2%) year undergraduate students. Half of the respondents (51.0%) enrolled in three or more units per semester. Variant forms of assessment and timeframes were used to assess students’ competence and understanding of the learning outcomes for their enrolled units. Majority of assessments were completed within a 24-hour window (48.2%), followed by those scheduled to be completed between 2 to 4 hours (38.7%).

The indicator scale used to measure academic integrity for alternative assessments was sufficiently reliable with a Cronbach alpha score of 0.77 (> 0.7). Response rate varied across academic integrity indicators (seeking help from peers, collaborating with others and seeking help from experts/ professionals/ senior colleagues). Although there were academic misconduct concerns, the results show that majority (> 68%) of students did not seek help from their peers, more than three-quarters (> 78%) did not collaborate with others, and more than four-fifth (> 80%) of students did not seek help from experts and therefore were not involved in these possible cheating behaviours (Table 1).

**Table 1:** Background characteristics of study sample and test of association between academic integrity indicators and study demographics

Variable	N (%)	Academic integrity indicators														
		Seeking help from peers					Collaborating with others				Seeking help from experts/professions/senior colleagues					
		Yes N (%)	No N (%)	NRR	*χ*^2^(df)	*p*-value	Yes N (%)	No N (%)	NRR	*χ*^2^(df)	*p*-value	Yes N (%)	No N (%)	NRR	*χ*^2^(df)	*p*-value
*Age*					**13.613 (4)**	**0.009**				3.155 (4)	0.532				0.091 (4)	0.999
18-24	112 (29.5)	32 (31.7)	69 (68.3)	9.8%			22 (21.8)	79 (78.2)	9.8%			20 (20.0)	80 (80%)	10.7%		
25-34	94 (24.7)	17 (20.2)	67 (79.8)	10.6%			14 (16.5)	71 (83.5)	9.6%			16 (18.8)	69 (81.2)	9.6%		
35-44	73 (19.2)	9 (13)	60 (87.0)	5.5%			11 (15.9)	58 (84.1)	5.5%			14 (19.7)	56 (80.0)	4.1%		
45-54	40 (10.5)	3 (7.9)	35 (92.1)	5.0%			4 (10.5)	34 (89.5)	5.0%			7 (18.4)	31 (81.6)	5.0%		
55 & above	11 (2.9)	2 (20)	8 (80)	9.1%			1 (10)	9 (90)	9.1%			2 (18.2)	9 (81.8)	0%		
(Missing)	50 (13.2)															
*Course of study*					**3.884 (1)**	**0.049**				**7.722 (1)**	**0.005**				0.538 (1)	0.463
Social Sciences	129 (33.9)	19 (15.2)	106 (84.8)	3.1%			12 (9.6)	113 (90.4)	3.1%			22 (17.6)	103 (82.4)	3.1%		
Nursing	209 (55.0)	45 (24.5)	139 (75.5)	12.0%			40 (21.6)	145 (78.4)	11.5%			39 (21.0)	147 (79.0)	9.6%		
(Missing)	42 (11.1)															
*Level of study*					1.490 (1)	0.222				0.109 (1)	0.741				0.001 (1)	0.979
Undergraduate	319 (83.9)	59 (20.2)	233 (79.8)	7.6%			49 (16.7)	244 (83.3)	7.3%			58 (19.7)	236 (80.3)	7.0%		
Postgraduate	16 (4.2)	5 (33.3)	10 (66.7)	21.1%			3 (20.0)	12 (80.0)	21.1%			3 (20.0)	12 (80.0)	21.1%		
(Missing)	45 (11.8)															
*Stage of study*					1.314 (3)	0.726				2.671 (3)	0.445				0.241 (3)	0.971
Year 1	111 (29.2)	23 (22.1)	81 (77.9)	6.3%			22 (21.0)	83 (79.0)	5.4%			21 (20.0)	84 (80.0)	5.4%		
Year 2	130 (34.2)	23 (19.7)	94 (80.3)	10,0%			15 (12.9)	101 (87.1)	10.8%			24 (20.5)	93 (79.5)	10.0%		
Year 3	75 (19.7)	12 (17.9)	55 (82.1)	10.7%			12 (17.6)	56 (82.4)	9.3%			12 (17.6)	56 (82.4)	9.3%		
Postgrad	22 (5.8)	6 (28.6)	15 (71.4)	4.5%			3 (14.3)	18 (85.7)	4.5%			4 (19.0)	17 (81.0)	4.5%		
(Missing)	42 (11.1)															
*Number of units enrolled*					5.318 (3)	0.150				0.367 (3)	0.947				1.110 (3)	0.775
1	62 (16.3)	11 (18.6)	48 (81.4)	4.8%			10 (16.9)	49 (83.1)	4.8%			11 (18.6)	48 (81.4)	4.8%		
2	80 (21.1)	9 (12.3)	64 (87.7)	8.8%			11 (14.9)	63 (85.1)	7.5%			12 (16.0)	63 (84.0)	6.3%		
3	81 (21.3)	17 (23.3)	56 (76.7)	9.8%			12 (16.4)	61 (83.6)	9.9%			15 (20.5)	58 (79.5)	9.9%		
4 or more	113 (29.7)	27 (26.0)	77 (74.0)	8.0%			19 (18.3)	85 (81.7)	8.0%			23 (22.1)	81 (77.9)	8.0%		
(Missing)	44 (11.6)															
*Time frame for alternative assessment*					n. a	n. a				n. a	n. a				n. a	n. a
2-4 hrs	147 (38.7)	31 (23.3)	102 (76.7)	9.5%			20 (14.9)	114 (85.1)	8.8%			25 (18.8)	108 (81.2)	9.5%		
24 hours	183 (48.2)	35 (20.5)	136 (79.5)	6.6%			26 (15.3)	144 (84.7)	7.1%			28 (16.3)	144 (83.7)	6.0%		
48 hours	48 (12.6)	6 (14.3)	36 (85.7)	12.5%			3 (7.1)	39 (92.9)	12.5%			9 (21.4)	33 (78.6)	12.5%		
1-2 week(s)	19 (5.0)	4 (25.0)	12 (75.0)	15.8%			3 (17.0)	14 (83.0)	10.5%			6 (46.0)	11 (64.0)	10.5%		
Other	61 (16.1)	9 (15.8)	48 (84.2)	6.6%			9 (15.8)	48 (84.2)	6.6%			10 (17.5)	47 (82.5)	6.6%		

*NRR* Nor-response rate

Bold *p*-values denote significant association at 5% level of significance

With regards to the nature of association between study demographics and academic integrity indicators, it was found that seeking help from peers was associated with age of students (*χ*^2^(4) = 13.631, *p* = 0.009) and course of study (*χ*^2^(1) = 3.884, *p* = 0.049). Students in the 18-24 age bracket often sought help from their peers (31.7%). The multivariable analysis revealed that students in this age brackets were at least 38% more likely to seek help from peers compared to the other age brackets (Table 2). Students pursuing nursing (25.5% [*n* = 45]) were found to seek help from peers compared to their counterparts from social sciences (15.2% [*n* = 19]). Nursing students were 21.5% more likely to seek help from peers compared to Social Science students. The majority of students who did not seek help from their peers, (84.8% [*n* = 106]) were enrolled in social sciences and (75.5% [*n* = 139]) were enrolled in nursing.

**Table 2:** Multivariable analyses of the association of demographic factors with propensity to commit assessment dishonesty such as seeking unauthorised help and collaboration

Variables	Seeking help from peers			Collaborating with others			Seeking help from experts/professionals		
	Est. (SE)	AOR (95% CI)	*p*-value	Est. (SE)	AOR (95% CI)	*p*-value	Est. (SE)	AOR (95% CI)	*p*-value
*Age*									
18 – 24	0	1	–	0	1	–	0	1	–
25 – 34	−0.480 (0.373)	0.619 [0.298, 1.286]	0.198	−0.259 (0.420)	0.772 [0.339, 1.759]	0.538	−0.004 (0.406)	0.996 [0.450, 2.206]	0.992
35 – 44	−0.958 (0.442)	0.384 [0.161, 0.912]	**0.030**	−0.226 (0.442)	0.797 [0.335, 1.896]	0.609	0.107 (0.422)	1.113 [0.487, 2.544]	0.800
45 – 54	−1.465 (0.677)	0.231 [0.061, 0.872]	**0.031**	−0.576 (0.640)	0.562 [0.160, 1.972]	0.368	0.230 (0.536)	1.259 [0.440, 3.599]	0.667
55 and above	−0.292 (0.905)	0.747 [0.127, 4.403]	0.747	0.773 (0.048)	0.475 [0.080, 1.257]	0.534	0.378 (0.889)	1.459 [0.256, 8.323]	0.671
*Course/Program*									
Social Sciences	0	1	–	0	1	–	0	1	–
Nursing	0.195 (0.357)	1.215 [0.603, 2.447]	0.586	0.941 (0.407)	2.562 [1.153, 5.690]	**0.021**	0.151 (0.352)	1.163 [0.584, 2.316]	0.668
*Level*									
Undergraduate	0	1	–	0	1	–	0	1	–
Postgraduate	1.112 (1.315)	3.040 [0.231, 39.990]	0.398	0.942 (0.784)	2.437 [1.542, 6.248]	**< 0.001**	0.600 (1.423)	1.821 [0.112, 29.635]	0.674
*Stage*									
Year 1	0	1	*–*	0	1	–	0	1	–
Year 2	−0.002 (0.371)	0.998 [0.482, 2.065]	0.995	−0.661 (0.411)	0.514 [0.231, 1.156]	0.108	−0.052 (0.371)	0.950 [0.459, 1.966]	0.890
Year 3	0.054 (0.456)	1.055 [0.432, 2.580]	0.906	0.004 (0.469)	1.004 [0.400, 2.516]	0.994	−0.453 (0.457)	0.635 [0.259, 1.557]	0.321
Postgraduate	−0.409 (1.206)	0.665 [0.063, 7.064]	0.735	−0.391 (0.859)	0.864 [0.214, 1.248]	0.958	−0.757 (1.275)	0.469 [0.039, 5.709]	0.553
*Number of units per semester*									
1	0	1	–	0	1	–	0	1	–
2	−0.342 (0.527)	0.710 [0.253, 1.995]	0.516	−0.141 (0.517)	0.869 [0.315, 2.393]	0.785	−0.182 (0.492)	0.834 [0.318, 2.188]	0.712
3	0.167 (0.498)	1.182 [0.445, 3.135]	0.738	−0.137 (0.528)	0.872 [0.310, 2.454]	0.795	0.294 (0.504)	1.342 [0.500, 3.602]	0.559
4 or more	0.353 (0.497)	1.423 [0.537, 3.770]	0.477	−0.144 (0.518)	0.866 [0.314, 2.388]	0.781	0.255 (0.494)	1.290 [0.490, 3.395]	0.606
*Duration*									
2-4 hours	−0.115 (0.378)	0.891 [0.425, 1.869]	0.760	−0.866 (0.468)	0.421 [0.168, 1.053]	0.064	−0.519 (0.399)	0.595 [0.272, 1.301]	0.194
24 hours	−0.246 (0.377)	0.782 [0.373, 1.638]	0.514	−0.816 (0.464)	0.442 [0.178, 1.097]	0.078	−0.746 (0.399)	0.474 [0.217, 1.036]	0.061
48 hours	−0.705 (0.513)	0.494 [0.181, 1.349]	0.169	−1.552 (0.715)	0.212 [0.052, 0.860]	0.030	0.096 (0.466)	1.100 [0.442, 2.741]	0.837
1 week	0.195 (0.930)	1.215 [0.197, 7.517]	0.834	0.164 (1.237)	1.179 [0.104, 13.318]	0.894	0.254 (0.930)	1.289 [0.208, 7.970]	0.785
2 weeks	0.175 (1.019)	1.192 [0.162, 8.784]	0.863	0.051 (1.030)	1.052 [0.140, 7.924]	0.961	1.426 (0.908)	4.162 [0.702, 24.669]	0.116
Other	−0.295 (0.491)	0.745 [0.285, 1.949]	0.548	−0.600 (0.573)	0.549 [0.179, 1.686]	0.295	−0.729 (0.503)	0.483 [0.180, 1.293]	0.147

Bold text denotes significant association at 5% level of significance

Categories with 0 estimates were used as reference levels in the modelling

*Est* Parameter estimate, *SE* Standard error, *AOR* Adjusted Odds ratio, *CI* Confidence interval

Additionally, an association was found between collaboration with others and course of study (*χ*^2^(1) = 7.722, *p* = 0.005). Again, higher proportion of students in the nursing programme (21.6%) collaborated with others to work on the alternative assessments compared to their counterparts from Social Sciences (9.6%). The adjusted odds ratio indicates that nursing students were 2 times more likely to collaborate with others compared to social science students (Table 2). Although, some proportion of students reported to have sought help from experts/ professionals/senior colleagues, no significant associations were found with the study demographics (*p* > 0.005) (see Table 1).

There was no significant difference between the stages of study and the percentage of students with academic integrity concerns. However, more than one fifth (> 20%) of first year students responded favourable to seeking help from their peers, collaborating with others, and seeking help from experts. Second year students were less likely to collaborate with others (12.9%) than seek help from peers and experts (> 19.7%). Twelve (< 17.9%) students in their third year indicated they asked for help from their peers, collaborating with others, and sought guidance from experts. Postgraduate students (28.6% [*n* = 6]) were more likely to seek help from their peers than collaborate or ask experts for help (see Table 1).

In terms of the duration of the assessment and academic integrity, it was found that higher proportions of students sought help from others when the duration for the assessment was greater than 4 h. For instance, for assessments of durations between 2 and 4 hours, nearly one-fifth of the students (18.8%) sought help from experts/professionals/senior colleagues compared to 46.0% for durations between 1 and 2 week(s). Notably, the upper bounds of the 95% confidence intervals of the adjusted odds ratio indicate that there is higher propensity for students to seek help from experts and professionals when assessment durations are relatively long (Table 2).

#### Qualitative findings

Thematic content analysis of the qualitative dataset resulted in aggregation of 274 findings into three themes and word cloud used to visualise the content of the qualitative dataset (Fig. 1). The themes focused on whether alternative online assessments made it easier for students to breach academic integrity. The themes are (i) impossible to cheat; (ii) easy to cheat and (iii) understanding the consequence of cheating. While some students believed that alternative assessment made it easier to cheat, others believed the structure of the *‘questions do not allow for cheating’* and that students are aware of the consequences of cheating. The probability of cheating depended *‘on the ethics of the student’*.

#### Impossible to cheat in alternative assessment

Majority of students perceived cheating was not easier in alternative assessments, they cited; *‘application of theory to practice’*, ‘*critical thinking requirement*’ in the assessments, ‘*limited timeframe*’ or ‘*time restrictions*’ there was no time ‘*to cheat nor think of ways of how to cheat!’, ‘availability of tracking devices’ ‘ethical standard*’, morality as some students ‘*value academic integrity and want to personally get the most out of the course and have marks that reflect own efforts*’ or ‘*comes down to each individual’s own integrity*’. Personal circumstance of students was also perceived as a deterrent for cheating in alternative assessments. For instance, ‘*being online student and not having friends*.

Well-designed assessment questions were indicated as a way of maintaining integrity in alternative assessments; ‘*cleverly structured questions*’ as ‘*without understanding the content it would have been impossible to answer the questions no matter the access to books*’ or ‘*if you don’t understand the information then you cannot apply the information obtained from course materials*’ and ‘*the questions required quite in depth answers*’. Other students were of the view that plagiarism detector software such as Turnitin and/or Cadmus prevented them from thinking about cheating; ‘*Turnitin would ensure if students collaborated it would be discovered*’ and ‘*as an open book exam you can’t really cheat unless you had someone else log in and do it for you’*. Some also felt the alternative assessment was ‘*an individualised exam. Not a group exam*’ so did not think of cheating.

A few students believed that with appropriate pre-assessment preparation by teaching staff, it was unlikely to cheat; ‘*students were well prepared for these assessments, therefore the likely hood (sic) of students cheating is minimal*’. On the part of students, ‘*proper preparation and organisation required no need for cheating as it was open book*’. Perceptions of students who strongly believed that the academic integrity was preserved, is summarised by one student as:

No, as the questions were structured differently so in the viva's you are asked in real time so you either know it or you don't. With my science exam the questions were structured very differently. You could not simply google the answer, I didn't use any of my note all I used was my textbook to give me some background knowledge so I could use that information to answer the question in my own words. It was submitted through turn it in (*sic*), so everything had to be your own work, the questions were very challenging and not basic questions they actually required researching just in order to answer the question

#### Easy to cheat in alternative assessment

For those who believed cheating was easier, they cited ‘*access to academic resources or notes or books’, ‘internet’ ‘google*’ and ‘*friendship or familiarity with course mates’ ‘too much allocated time for assessment*’ and ‘*being an on-campus student with friends*’ as the main reasons for cheating. Collusion and collaboration were cited as the main forms of cheating in the alternative assessments. Cheating was perceived to be prevalent among students who ‘*believe that they have not had sufficient support in their studies’*. Some students noted that it was easy for them to ‘*message for answers or get perspective’* ‘*discussed with colleagues in the library’ ‘a phone call with other students discussing answers’* or ‘*easily share information using social media as nobody is invigilating*’ or ‘*yes because there is no invigilation to stop people from collaborating*’ **or** ‘*anyone could be undertaking the exam on their [student’s] behalf*’. Some students were of the view that cheating was common in ‘*generally poorly written*’ assessment. One student stated ‘*Yep, everyone did. There were a whole range of social media formats to share answers*’.

Having friends or close classmates was also considered a facilitator for cheating in alternative assessments: ‘*I guess so, if you had a group of friends that where [sic] in the same class you could probably meet up and do it together*’ and ‘*I don’t know any of my peers in my units[subject or course] so I had no one to collaborate with’*. It was perceived that, academic integrity was hard to maintain in alternative assessments that were not well designed:

‘I would say that it's probably better to design an assessment that requires explanation rather than google-able answers.’ ‘For facts you could google or multiple-choice questions that come straight from Quizlet, absolutely.’

‘100% easier for students to cheat. If they wanted students could google answers, read answers straight from the textbook, be facetiming friends asking for answers, I heard someone say they used 'google home' to search for answers for them, getting together in groups to sit the assessments.’

#### Understanding the consequence of cheating

Students were aware of the consequence of cheating as all students have completed the University’s academic integrity course. ‘*Completing the academic integrity course, I believe everyone did their assessments independently without collaborating or contacting others*’ as such ‘*all students are aware of the consequences involved with academic misconduct and would not want to jeopordise (sic) their studies by doing the wrong thing*’. Consequences were also narrated as; ‘*cheating eventually catching up on them in future’ ‘cheating on themselves’, ‘the disservice they are doing will come back to haunt them*’, Understanding the consequences and outcomes of academic integrity also served as a demotivation for cheating in alternative assessments. The consequence of academic misconduct in future practice was narrated by one student as:

‘I think to a degree there isn't a problem with looking up elements of the assessment as you go, but it is problematic if someone went in to practice without having put in the effort to truly learn and grasp certain elements of the unit/subject content’

## Discussion

The COVID-19 crisis has significantly impacted the higher education sector. Social distancing protocols and the conflicting need for continuity of education and the necessity to contain the spread of the virus meant that universities shift their operations online (Rashid and Yadav 2020). The shift to the online mode in teaching and learning presented systemic challenges, allowing little time for university administrators to conduct in-depth risk assessments of academic integrity (Slade and Benson 2020). This study provided a serendipitous opportunity to examine the practicality and implications of alternative assessment and non-invigilated examinations and tests *vis-a-vis* academic integrity in higher education.

Thus, the purpose of the current study was to explore the perceptions of university students on academic integrity concerns associated with alternative and non-invigilated assessments, implemented during the COVID-19 pandemic. Findings from the study suggest that on the average, more than three quarters (> 75%) of students did not engage in any of the three cheating behaviours associated with alternative assessments. This figure correlated well with the qualitative findings that alternative assessments are hard to cheat. For the nearly 25% students that perceived one or more cheating behaviours in alternative assessments, there was increased risk of cheating among younger people (18-24 years) and nursing students. The study also found that the longer the duration of the assessment, the higher the risk of cheating. Qualitative evidence suggests that while majority of students perceived that alternative and non-invigilated assessments are hard to cheat, few students believed that it is easy to cheat in these assessments but, at the same time, students were aware of the current and future implications of academic misconduct.

Findings from our study showed that majority of students did not engage in cheating behaviours in the alternative assessments. Furthermore, most students did not perceive cheating to be easy in alternative assessments. The data showed that although some students did not cheat out of personal and ethical values, nearly one fourth of students accessed unauthorised resources, colluded and collaborated with their mates or sought help from senior colleagues or experts. This figure is high compared to other studies with traditional invigilated on-campus assessments and online assessments of 8 and 12% respectively (Harris et al. 2020; McCabe 2005). When age is controlled to 25 years or less, our data shows high perception of academic misconduct (24.5%) in online alternative assessments than the 12% previously reported by Harris et al. (2020) but contrary to that of McCabe (2005). McCabe (2005) study shows that, with written assignments, 24% of undergraduate students are likely to “receive unpermitted help from someone on an assignment” while 42% will “work with others on an assignment when asked for individual work” (otherwise known as collusion). Perhaps, the use of academic misconduct tools and design of questions and other strategies in our study may have contributed to the observed reduced risk of collusion.

In addition, the study revealed that the number of units/subjects enrolled per semester is positively correlated to cheating behaviours. This can be explained in the context of the COVID-19 related stress and the increased workload associated with more units. This finding is consistent with previous studies that found stress and increased workload to be predictors of academic misconduct in higher education (Amigud and Lancaster 2019; Tindall and Curtis 2020).

Our study revealed that young people (18-24) perceived high academic misconduct in alternative assessments than older people. This could be because these young people, considered Generation Z, rely heavily on the internet/technology and friends/peers for information and support respectively. Generation Z are fully aware of the different ways cheating can occur in online non-invigilated assessment than older generations (Hernandez-de-Menendez et al. 2020). Our finding is similar to that of Kisamore et al. (2007) and Landa-Blanco et al. (2020) where they reported that older people are less likely to perceive or commit academic misconduct than younger people. Our findings also suggest that many technologies such as mobile phone messaging and social media platforms were used to aide cheating in alternative assessments. These platforms are highly patronised by Generation Z and are frequently used for cheating in alternative assessments. In this regard, education on academic ethics and the repercussion of cheating will be very critical in a post COVID-19 academic integrity policy and praxis (Curtis et al. 2022; Morris 2018). Additionally, academic integrity roles should be well-resourced and supported within the bureaucracy of academic institutions (Eaton 2020; Seeland 2020). Furthermore, existing academic misconduct detection and management architecture will need to be reviewed if not overhauled as most of them appear to cater for face-to-face learning mode, aside from inconsistencies in their application (Amigud and Pell 2021).

Like age, there was a negative relationship between undergraduate academic year and cheating behaviours. Students in first year were more likely to be involved in cheating behaviours than students in second and third years. This could result from first year students’ inability to transition effectively into higher education before the drastic changes in assessments as a result of COVID-19. These students may not have had the full opportunity of academic integrity training compared to their senior colleagues due to the chaos created by COVID-19. With COVID-19 restrictions, academic institutions, the gate keepers of academic integrity, had to juggle between organisational adjustments and learning and delivery approaches. As a result, academic integrity measures were not the priority for universities who had to focus on building their online systems to meet the unprecedented demands for online teaching and learning (Slade and Benson 2020). This finding may be explained in the work of Bretag et al. (2014), in which over 80% of students responded that the first time they learnt about academic integrity was during their university orientation. It is recommended that innovative approaches to academic integrity workshops be implemented for first year students who are likely to be Generation Z to develop a culture of academic integrity.

Of the three academic misconduct behaviours explored in alternative assessments, our study showed that collusion is the commonest form of cheating with more than 31% of students confirming that they have sought help from peers. This behaviour is perceived to be common among on campus students with friends or classmates that they can collaborate through other means such as phone text messaging or social media platforms. As reported in other studies (Harris et al. 2020), this form of cheating is the commonest in project-related or assignment type assessments. Perhaps, the possible reason for this observation is students’ perception of collusion being a form of team work or supporting each other to succeed (Forkuor et al. 2019). In our study, although students were aware of the requirement to undertake the assessment individually, collusion remained prevalent. The emphasis on team and collaborative work in nursing education/profession (Barton et al. 2018), may have contributed to the high rate of collusion among nursing students. Ongoing education on the differences between collusion and unauthorised collaboration must be the focus in designing academic integrity interventions in courses where teamwork is highly regarded.

Furthermore, our study also found positive correlation between assessment time and risk of academic misconduct. The risk of academic misconduct increases by more than double when the assessment duration is 1-2 weeks compared to 2-4 hours. The longer the assessment duration, the more likely students are to collude or collaborate with others. It is recommended that assessment be given limited time duration—not more than 4 hours—to minimise this risk.

The qualitative data in the current study suggested ways in which risk of academic integrity can be minimised if not eliminated in alternative assessments. Aside from the promotion of ethical values among students, making assessments time-bound (less than 4 h) and use of plagiarism detection softwares were mentioned in the data and corroborated in other studies as useful measures for bolstering academic integrity in a non-invigilated assessment setting (Brown and Janssen 2017; Denisova-Schmidt, 2017). Other ideas touched on the need to design assessments that require application of theory to practice as opposed to “google-able” questions and assignments. This finding is consistent with the work of Golden and Kohlbeck (2020) in which they reported decreased rate of cheating when online question banks are paraphrased. Ongoing education on present and future consequence of academic misconduct should be a usual conversation between academics and students as this has proven to minimise the risk of academic misconduct in alternative assessment.

## Limitations and future research

In self-reported questionnaires, there is the tendency for fake responses, typically presenting favourable images of phenomenon under investigation, referred to a social desirability bias. This bias can confound study results by creating false relationships or obscure true relationships (Van de Mortel 2008). In this study, we observed an average non-response rate of approximately 15% across the indicator scales for academic integrity. This indicates that students who were not comfortable with the indicator items opted not to answer. It can be inferred that the majority of students responded honestly to the questionnaire items, and thus the conclusions arrived at are meaningful and informative to instigate appropriate measures to safeguard academic integrity in alternative assessments.

Another limitation of the study is related to the sampling method—convenience sampling—which may be reflective of the nonresponsive bias and the inclusion of only nursing and social science students. Students who do not feel strongly for or against the alternative assessment did not feel the need to complete the survey (Wang & Cheng, 2020). This may result in a limitation to generalisability of the results for all students. Future research should explore the academic integrity concerns associated with alternative assessments from the perspective of both academics and all students from various courses to present a holistic picture.

## Conclusion

This paper sought to explore the perceptions and experiences of academic integrity concerns of 380 tertiary students in higher education regarding online alternative assessments during the COVID-19 pandemic. The study employed a cross-sectional mixed method design. We conclude that alternative assessment is a viable option for assessing students if measures are implemented to reduce associated academic integrity risks. Like other forms of traditional assessments, alternative assessments have risk of academic integrity breach; however, with the right strategies, they could serve as effective means of assessing learning outcomes. In this study, although cheating was not found to be a generality among participants, some students reported consulting unauthorised sources and colluding with their peers. Addressing such vices in tertiary institutions would require continuous education of students on the importance of academic integrity as well as the costs of academic dishonesty to the university, the students, and their future. Other measures, such as designing time-bound assessments, non-googleable questions, use of plagiarism detection softwares and designing assessments that require application of theory to practice would go a long way to making academic dishonesty less popular with alternative and non-invigilated assessments. COVID-19 and its attendant issues with academic misconduct has brought to the fore the need for universities to resource its academic integrity gate keepers by training academics on developing assessments that can enhance academic integrity.

## Supplementary Information

**Additional file 1.** Survey for academic integrity.

## Data Availability

Data is available upon request.

## Abbreviations

COVID-19: Coronavirus Disease-19
ID: Identification card for identifying students
