# Supplementary material for: COVID-19 and alternative assessments in higher education: implications for academic integrity among nursing and social science students
Source: Int J Educ Integr. 2023 May 1;19(1):8. doi: 10.1007/s40979-023-00129-0 (PMC10149154; doi:10.1007/s40979-023-00129-0)
Supplement: Supplementary file 1 — Additional file 1. Survey for academic integrity. [file 40979_2023_129_MOESM1_ESM.docx]

**Additional file 1: Survey for academic integrity**

Survey: Alternative assessments and implications for academic integrity

Start of Block: Consent form

Q1 **CONSENT**

- Yes
- No

Skip To: End of Survey = No

End of Block: Information letter and consent form

Start of Block: Demographic data

Q2 How old are you?

▼ 18-24 (1) ... 75 and above (10)

Q3. Which course are you currently enrolled in?

- ________________________________________________

Q4. Are you enrolled in an undergraduate course or postgraduate course?

- ________________________________________________

Q5. Which stage of your course are you enrolled in?

- ________________________________________________

Q6. In 2020 Semester 1, how many units did you undertake a take-home alternative assessment?

- 1 (1)
- 2 (2)
- 3 (3)
- 4 (4)
- 5 or more (5)

Q7. Please indicate the timeframe you were allocated to complete the alternative assessment, once opened?

- 2-4 hours (1)
- 24 hours (2)
- 48 hours (3)
- 1 week (4)
- 2 weeks (5)
- Other please specify

Start of Block: Academic integrity

Q8. Did you defer your end of semester alternative assessment?

- Yes (6)
- No (8)
- Not applicable (7)

Q9. Did you defer your end of semester alternative assessment?

- Yes (6)
- No (8)
- Not applicable (7)

Q10. If yes, why?

________________________________________________________________

Q11. If yes, did you discuss the content of the assessment with anyone who had already taken the alternative assessment?

- Yes (12)
- No (13)

Q12. If so, did you find it helpful?

- Yes, please explain below (1) __________________________________________________
- No, please explain below (3) __________________________________________________

Q13. How often did you undertake any of the following during your alternative assessment? I frequently;

|  | Agree (19) | Disagree (20) |
| --- | --- | --- |
| Seeking help from peers (1) |  |  |
| Collaborating with others (2) |  |  |
| Seeking help from experts/professional/ senior colleagues (3) |  |  |

Q14. It was easy for me to seek academic help from peers and/or others.

- Agree (1)
- Disagree (2)

Q15. Do you think the alternative assessment made it easier for students to cheat? Please explain your response

________________________________________________________________

End of Block: Academic integrity
